# Automated detection of third molars and mandibular nerve by deep learning

**DOI:** 10.1038/s41598-019-45487-3

**Published:** 2019-06-21

**Authors:** Shankeeth Vinayahalingam, Tong Xi, Stefaan Bergé, Thomas Maal, Guido de Jong

**Affiliations:** 1 Department of Oral and Maxillofacial Surgery, Radboud University Nijmegen Medical Centre, Nijmegen, The Netherlands; 2 Department of Neurosurgery, Radboud University Nijmegen Medical Centre, Nijmegen, The Netherlands; 3 Radboudumc 3D Lab, Radboud University Medical Centre, Nijmegen, The Netherlands

**Keywords:** Translational research, Outcomes research

## Abstract

The approximity of the inferior alveolar nerve (IAN) to the roots of lower third molars (M3) is a risk factor for the occurrence of nerve damage and subsequent sensory disturbances of the lower lip and chin following the removal of third molars. To assess this risk, the identification of M3 and IAN on dental panoramic radiographs (OPG) is mandatory. In this study, we developed and validated an automated approach, based on deep-learning, to detect and segment the M3 and IAN on OPGs. As a reference, M3s and IAN were segmented manually on 81 OPGs. A deep-learning approach based on U-net was applied on the reference data to train the convolutional neural network (CNN) in the detection and segmentation of the M3 and IAN. Subsequently, the trained U-net was applied onto the original OPGs to detect and segment both structures. Dice-coefficients were calculated to quantify the degree of similarity between the manually and automatically segmented M3s and IAN. The mean dice-coefficients for M3s and IAN were 0.947 ± 0.033 and 0.847 ± 0.099, respectively. Deep-learning is an encouraging approach to segment anatomical structures and later on in clinical decision making, though further enhancement of the algorithm is advised to improve the accuracy.

## Introduction

The removal of the third molar is one of the most frequently performed surgical procedures in oral surgery. In the United States 10 million third molars are removed from approximately 5 million people annually^1^ Across the past three generations, the worldwide rate of third molar impaction was hovering at around 24.4%^2,3^.

As with other forms of surgery, the surgical removal of the lower third molars is associated with risk for complications. One of the most distressing complications following the removal of lower third molars is damage to the inferior alveolar nerve (IAN)^4^. IAN injuries cause temporary, and in certain cases, permanent neurosensory impairments in the lower lip and chin^5^, with an incidence of 3.5% and 0.91% respectively^6^. Therefore, preventing damage to the IAN is of utmost importance in the daily clinical practice.

Conventional two-dimensional panoramic radiograph, the orthopantomogram (OPG), is the most commonly used imaging technique to assess third molars and their relationship to the mandibular canal^7–10^. Previous studies have demonstrated that certain radiographic features on OPGs, such as darkening of the root, narrowing of the mandibular canal, interruption of the white line, are risk factors for IAN injuries^11–13^. A recent meta-analysis reported that darkening of the roots had a high specificity in predicting IAN injury^14^. However, the predictive factor was not satisfactory as an evident heterogenicity was seen across all the included studies. Apparently, high intra-observer and inter-observer variability of the radiographic signs exists. Examiners are not always able to identify and assess the specific signs in a reliable way on OPGs^8^.

Prediction and evaluation modelling methods based on deep-learning have proven to be useful in solving complex multifactorial problems in medicine^15^. Deep-learning has been applied to identify pulmonary nodes on high-resolution CTs and state-of-the-art performance has been achieved^16^. Parallel, there may be a high potential for the implementation of deep-learning in the detection of third molars, mandibular canals and the identification of certain radiographic signs for potential IAN injuries. The combined use of deep learning and OPG may allow an improved risk assessment of IAN injuries prior to the removal of third molars.

The aim of this present study was to achieve an automated high-performance segmentation of the third molars, and the inferior alveolar nerves (IAN) on OPG images using deep-learning as a fundamental basis for an improved and more automated risk assessment of IAN injuries.

## Results

### Lower M3 dentition detection

The lower M3 had a mean DICE of 0.947 (SD = 0.049) for the training data and a DICE of 0.936 (SD = 0.025) for the validation data (Table 1). In the training data one M3 was missed. Although a mean lower M3 was composed of less than 1% of the total OPG pixels, a mean and median sensitivity of 95% was achieved in both the training and validation set (Fig. 3).

**Table 1:** DICE-coefficients of third molar and inferior alveolar nerve training and validation data.

Dataset	Dice coefficient				Jaccard index			
	Mean	Median	SD	5–95% Perc.	Mean	Median	SD	5–95% Perc.
M3 Training	94.7%	95.3%	4.9%	91.8–97.4%	90.3%	91.1%	6.8%	84.9–94.9%
M3 Validation	93.6%	93.4%	2.5%	89.4–96.9%	88.1%	87.6%	4.4%	80.8–93.9%
IAN Train	76,8%	78,9%	11,9%	53,3–91,2%	63,8%	65,2%	14,5%	36,4–83,9%
IAN Validation	80,5%	85,6%	10,8%	58,4–90,1%	68,7%	74,8%	14,0%	41,2–82,0%
**Dataset**	**Sensitivity**				**Specificity**			
	**Mean**	**Median**	**SD**	**5–95% Perc**.	**Mean**	**Median**	**SD**	**5–95% Perc**
M3 Training	95.4%	96.5%	5.4%	91.8–98.0%	99.9%	100.0%	0.0%	99.9–100.0%
M3 Validation	94.7%	95.0%	3.3%	88.9–98.6%	99.9%	99.9%	0.0%	99.8–100.0%
IAN Train	83,8%	86,6%	13,2%	58,2–98,8%	96,0%	96,2%	2,2%	91,9–99,3%
IAN Validation	84,7%	86,5%	9,9%	67,1–95,4%	96,7%	97,5%	2,5%	91,6–99,3%

Mean, median, standard deviation, and 5–95% percentiles of the DICE coefficient, jaccard index, sensitivty and specificity of the segmentations per structure/dataset.

### IAN detection

The IAN detection had a mean DICE of 0.768 (SD = 0.119) for the training data and a DICE of 0.805 (SD = 0.108) for the validation data (Table 1). Median values for training and validation data were slightly higher than the mean values, 0.789 and 0.856 respectively. The IAN detection scored the lowest of all the detection networks; a mean DICE of well above 0.75 was obtained. Optical inspection showed three types of position agreements between the automatically and manually segmented IAN: good segmentations had only minor flaws and none of them around the third molar (root) region, while mediocre cases had greater position disagreements and the third molar region was largely unaffected, and the group of poor segmentations showed inaccurate segmentation of IAN in the third molar region (Fig. 4). Considering the optical inspection as well as a sensitivity of 0.838 (SD = 0.132) for training data and 0.847 (SD = 0.099) for validation data, and a median of 0.866 and 0.865 respectively, the automated segmentations by using U-net were mostly satisfactory.

## Discussion

Deep learning algorithms are evolving and are being increasingly applied in different medical fields, mainly to detect and segment clinically relevant anatomical structures or pathological changes, such as cancer^17^, tuberculosis^18^ or skin lesion^19^. However, the application of deep-learning in oral and maxillofacial surgery and dentistry is relatively scarce. Only the preliminary use of deep-learning in different topics such as caries detection or automatic dental radiography analysis has been described^20^.

Deep-learning is consisted of convolutional neural networks that is successfully applied to analyse visual imagery. CNN networks are able to detect and segment certain patterns in a large data set, such as a 2D radiograph or 3D CT scan. CNN can identify a group of pixels or voxels that make up either the contour or the interior of objects of interest. By changing certain characterises of the CNN architecture, the way of automatic segmentation can be adjusted to detect certain voxel patterns in a volume of interest^15^.

One of the most cited segmentation CNN applied in the medical field is the “U-net”. U-net has a simple and clear architecture and is aimed particularly at segmenting osseous and soft tissue structures. Compared to other CNNs, the accuracy of segmentation U-net is significantly better^21^. Ronneberger applied U-net to segment the enamel, dentine and pulp on dental radiographs (bitewings) and obtained a mean dice-coefficient of 0.56^21^. In some cases, a dice-coefficient of 0.70 was obtained, indicating the potential of further improvements of the U-net architecture.

The potential of applying U-net in the automated segmentation of third molars and IAN is investigated in the present study. By changing the U-net architecture and improvements in the training of U-net in performing segmentations, encouraging results have been obtained in both the identification and segmentation of third molars and IAN. However, in some cases, U-net was unable to locate and segment both predetermined anatomical structures satisfactorily. Several factors are associated with the underperformance. Firstly, the lack of contrast on the OPGs between the mandible and the mandibular canal complicated the task of segmentation for both the observer and the CNN. OPGs do not have constant image intensity in the region of the mandibular canals. Secondly, the mandibular canal varies significantly in its shape and location among patients^17^. Thirdly, since only one segmenting observer and one correcting observer segmented the OPGs only once a certain degree of interobserver and interobserver variability might occur leading to a potentially lower DICE-coefficient. The OPGs were initially scaled and cropped to a lower resolution than the native OPG resulting in a loss of data which can be useful for segmentation. Due to the high DICE-coefficient for both the overall dentition and M3s as well as the use of the near native resolution for the IAN with a lower DICE it seems that the resolution might not be a severe issue on the performance of the segmentation. In case the OPGs would be analysed in full detail it is possible to resort to more powerful PC’s or use the overlap-title strategy as originally used for the U-nets^21^.

There are several approaches to improve the segmentation of third molars and the IAN in particular. The first method to counteract the variety in shape and boundary on native images is by minimizing the region of interest. It is not necessary for the CNN to segment the complete mandibular canal. The only essential area for the risk assessment is the region of the third molar and its roots. The second suggestion is to increase the training data set with annotated OPGs. Although the applied augmentation due to rotation resulted in a high-performing segmentation in case of third molar, the accuracy for the IAN remained to be lower. The increase of annotated OPGs may lead to a better overall performance^21^. Thirdly, the use of DICOM file format for OPGs can enhance the segmentations. In this study, the OPGs were exported from the electronic patient files in JPEG files exports, resulting in 256 greyscale values with 8-bit per channel. An alternative to this file format is DICOM, which can natively hold a much larger range of greyscale values. Using these native DICOM files can potentially result in a higher contrast, making the IAN better to segment both manually and automatically. Another approach towards an enhanced IAN segmentation could be the use of cone-beam CT (CBCT) scans instead of OPGs. Among other segmentation techniques U-nets can also be applied to 3D volumetric shapes like CBCT-scans^22^. The CNN used in this study is specialized in segmentation based on shape. Yet, this CNN has another beneficial characteristic of taking topography into consideration. The problematic point of shape and boundary could also be solved when 3D data sets are provided, as the nerve appears as a circular cross-section in a CBCT scan. It could be possible that the 3D U-nets natively provide good segmentation in CBCT scans or that they yield the same shape and topology properties when used with double inputs. In this way, the anatomical positional relationship between the IAN and M3 could be evaluated. The main drawback of such an approach is the patient´s higher exposition to radiation.

It should be noted that alternative segmentation techniques exists to perform similar segmentations, such as watershed segmentation^23^, canny based segmentation^24^ or random forest regression voting constrained local models^25^, which were introduced as segmentation algorithms for OPGs in the past^26–28^. Since this study is more observational by character, comparative studies are required in the future, in order to the quality of different segmentation methods, especially semantic segmentation^29,30^, before the clinical implementation.

The encouraging results obtained from the present study in the segmentation of third molars and the IAN, was the first step on the way to a successfully implement deep-learning in daily clinical practice. The exact location and shape of the third molar roots in relation to the mandibular canal has to be determined accurately and in a reproducible way. Also risks patterns need to be identified. Individual risks need to be attributed to each factor and the CNN has to be able to sum up all the risk factors and the associated risks to give one final risk. Nevertheless the usage of deep-learning might not only provide professionals with additional information to optimize treatment planning and risk identification, but also automatically improve dental radiographs by dismissing unnecessary artefacts. The greatest advantage of deep-learning is the improvement in diagnostic without changing the present infrastructure and the wide range of application. For instance, the implementation of deep learning in caries management could enhance early caries detection and optimize the moment of intervention, thereby reduce the increasing global health burden induced by dental caries.

## Material and Method

This study was conducted in accordance with the World Medical Association Declaration of Helsinki on medical research ethics. The approval of this study was waived by the Institutional Review Board (CMO Arnhem-Nijmegen) and informed consent were not required as all image data were anonymized and de-identifed prior to analysis (decision no. 2019–5232). All methods were carried out in accordance with relevant guidelines and regulations.

### Patients

Orthopantomograms (OPGs) of patients who were admitted at the Department of Oral and Maxillofacial Surgery of Radboud University Nijmegen Medical Centre in 2017 were randomly selected. The inclusion criteria were the presence of at least 12 adjacent teeth with at most one missing element in between, at least one lower third molar and a minimum age of 16. Permanent fixed lingual retention wires and/or fillings are quite common in the (Dutch) population so OPGs containing these were not excluded to give a good societal representation. Blurred and incomplete OPGs were excluded from further analyses.

The collected data were de-identified and anonymized prior to analysis. Patients who signed the opt-out for anonymized research were also excluded conform the institution’s policy. Digital panoramic radiographs were acquired with a Cranex Novus e device (Soredex, Helsinki, Finland), operated at 77 kV and 10 mA, using a CCD sensor. The OPGs were taken with patients standing upright, head supported by the 5-point head support, with the upper and lower incisors biting gently into the bite block. A total of 81 OPGs were analysed.

### M3 and IAN detection workflow

**Figure 1:**
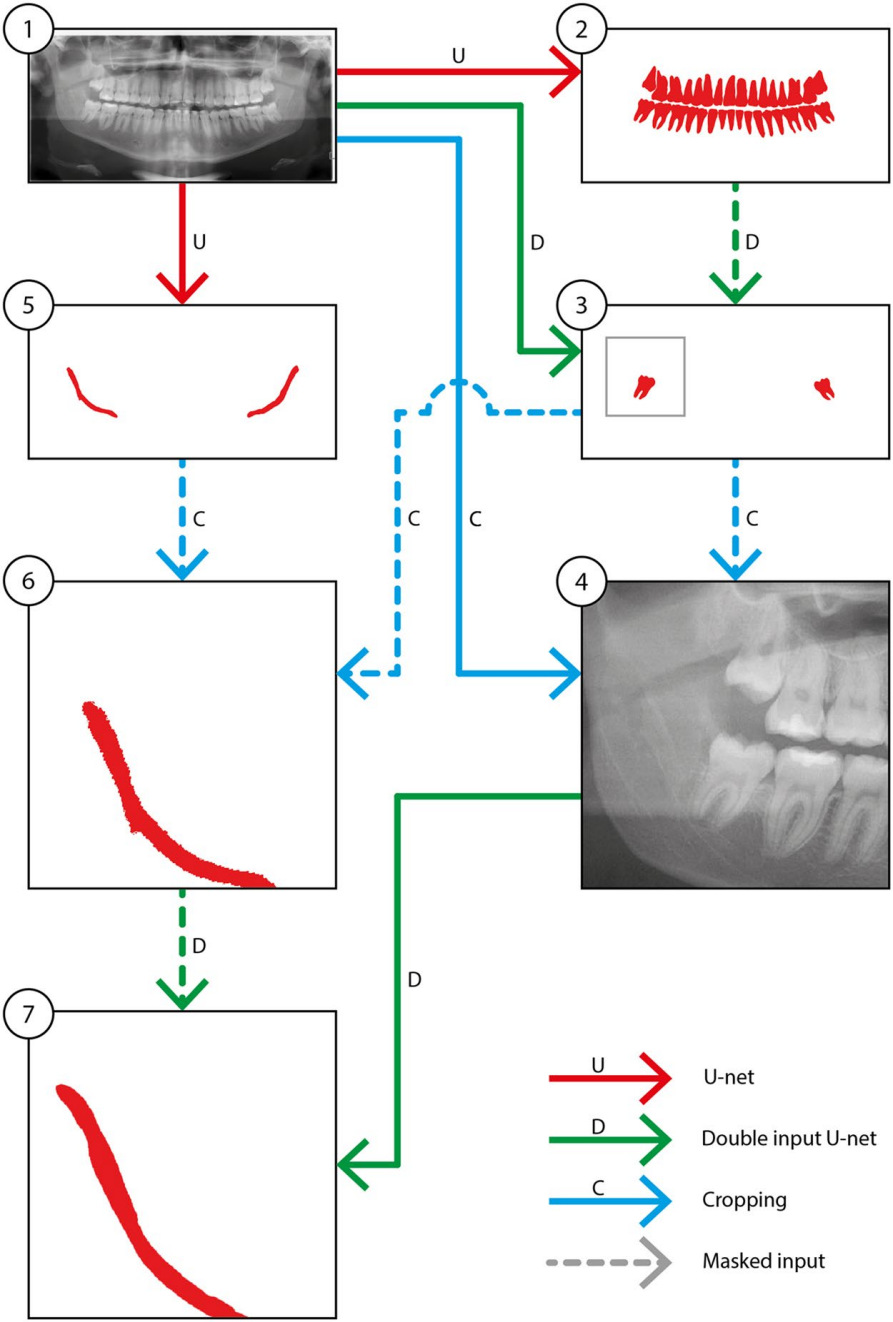
The workflow of third molars and inferior alveolar nerve segmentation process.

A workflow for the M3 detection and nerve segmentation was created using image processing and deep-learning networks (Fig. 1). In depth details of each step are described in the appendix. The core mechanic of the detection revolves around a deep-learning segmentation network called a U-net which is used in the subsequent steps^21^. The initial step 1 is the image acquisition and data pre-processing. This step prepares the data for standardizing the size and applying contrast enhancement to the OPG (Fig. 1). This is followed by the overall dentition detection (step 2) by providing the pre-processed OPG to a 6-layer deep-learning U-net. The lower M3 detection is executed in step 3 using the same pre-processed OPG from step 2 as well as the result (detected dentition mask) of step 2 by providing these to a specifically designed double input 6-layer U-net. Using the detected third molars in step 3 an automated crop of the original OPG is made in the fourth step 4. A margin extending caudally around the M3 is taken from the OPG to include a part of the IAN. Furthermore, the pre-processed OPG is also used to make a rough (low accuracy) extraction of the IANs in step 5 with a 5-layer U-net. The same cropped area of the original OPG around the third molar as taken in step 4 is also taken of the rough IAN segmentation of step 5 resulting in a cropped IAN for step 6. Finally in step 7 the refined IAN is detected in the crop from step 4 and step 6 using a 6-layer U-net. These steps result in the overall dentition (step 2), the isolated third molars (step 3) and the isolated IANs (step 7) which can be used for further analysis.

### Deep-learning network training

Each step holding a Deep-Learning network (Steps 2, 3, 5 and 7) has been trained using annotated data. Training data were obtained by manual segmentations as well as the results of running the manual segmentations through the workflow. The specific training conditions and rules can be found in the Appendix (see supplementary data).

**Figure 2:**
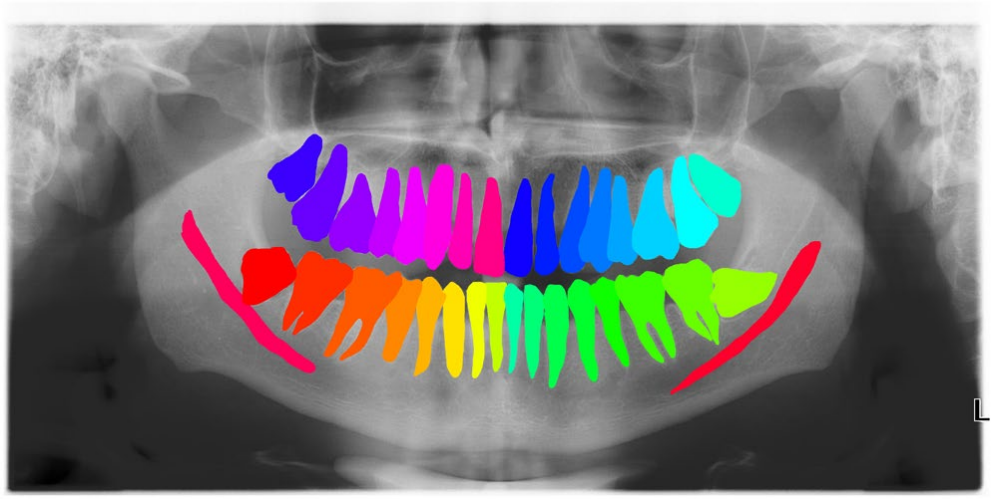
Color-coded OPGs with manually segmented teeth and inferior alveolar nerve.

For the training data all present teeth in the maxilla and mandible, and the mandibular canal, were manually segmented one by one on OPGs. Each segmented tooth and mandibular canal was attributed with a distinct colour, and labelled according to the FDI tooth numbering system with Adobe Photoshop CC 2017 (Adobe System Incorporated, San Jose, California) by one observer (SV) (Fig. 2). A second observer (TX) checked the segmentations and made further refinements. The color-coded OPGs images were uniformly scaled and cropped to 2048 by 1024 pixels using Adobe Photoshop CC 2017.

**Figure 3:**
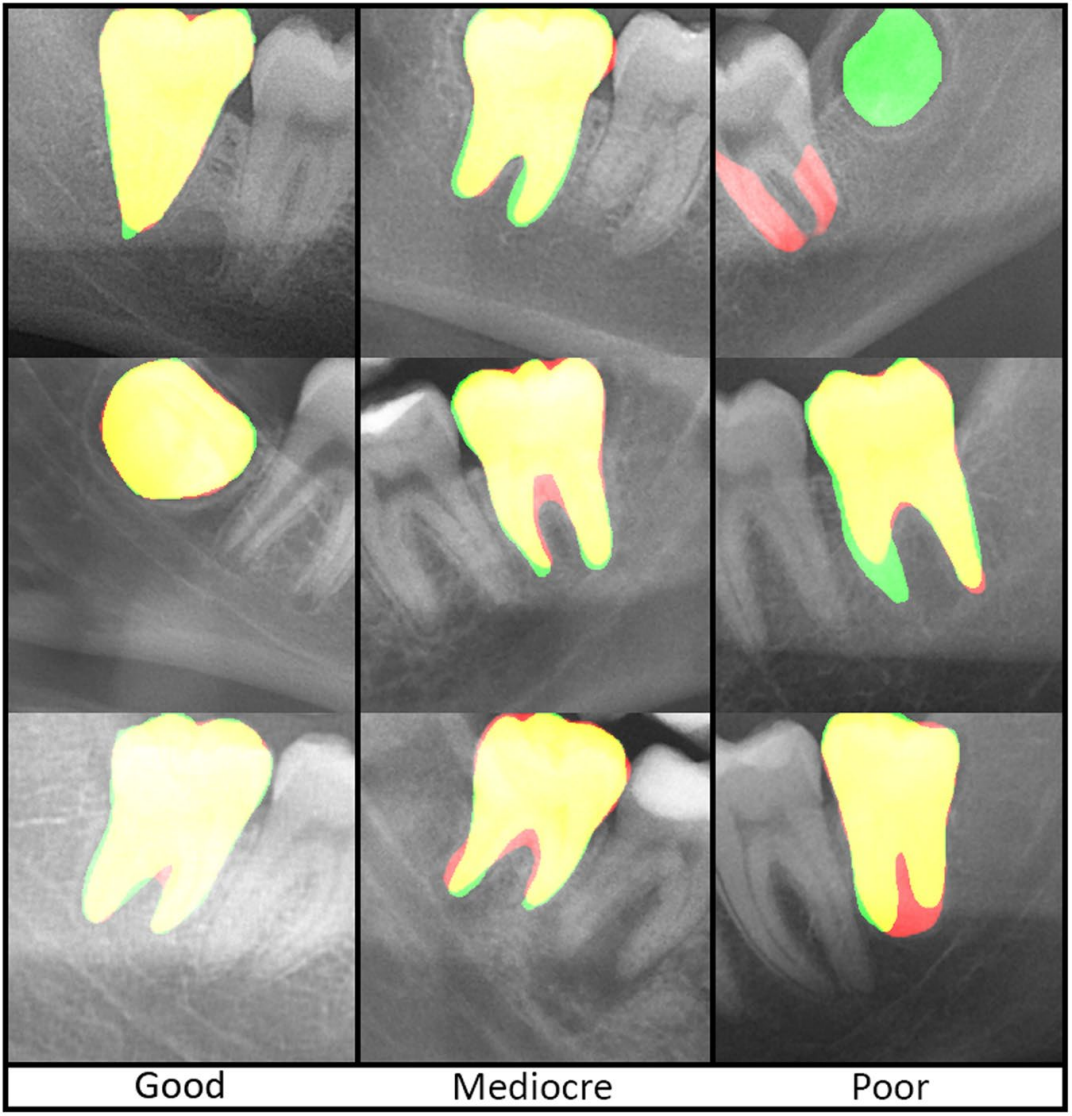
Overview of third molar segmentations. Green: manual segmentation, red: automatic segmentation, yellow: overlap between automatic and manual segmentation.

**Figure 4:**
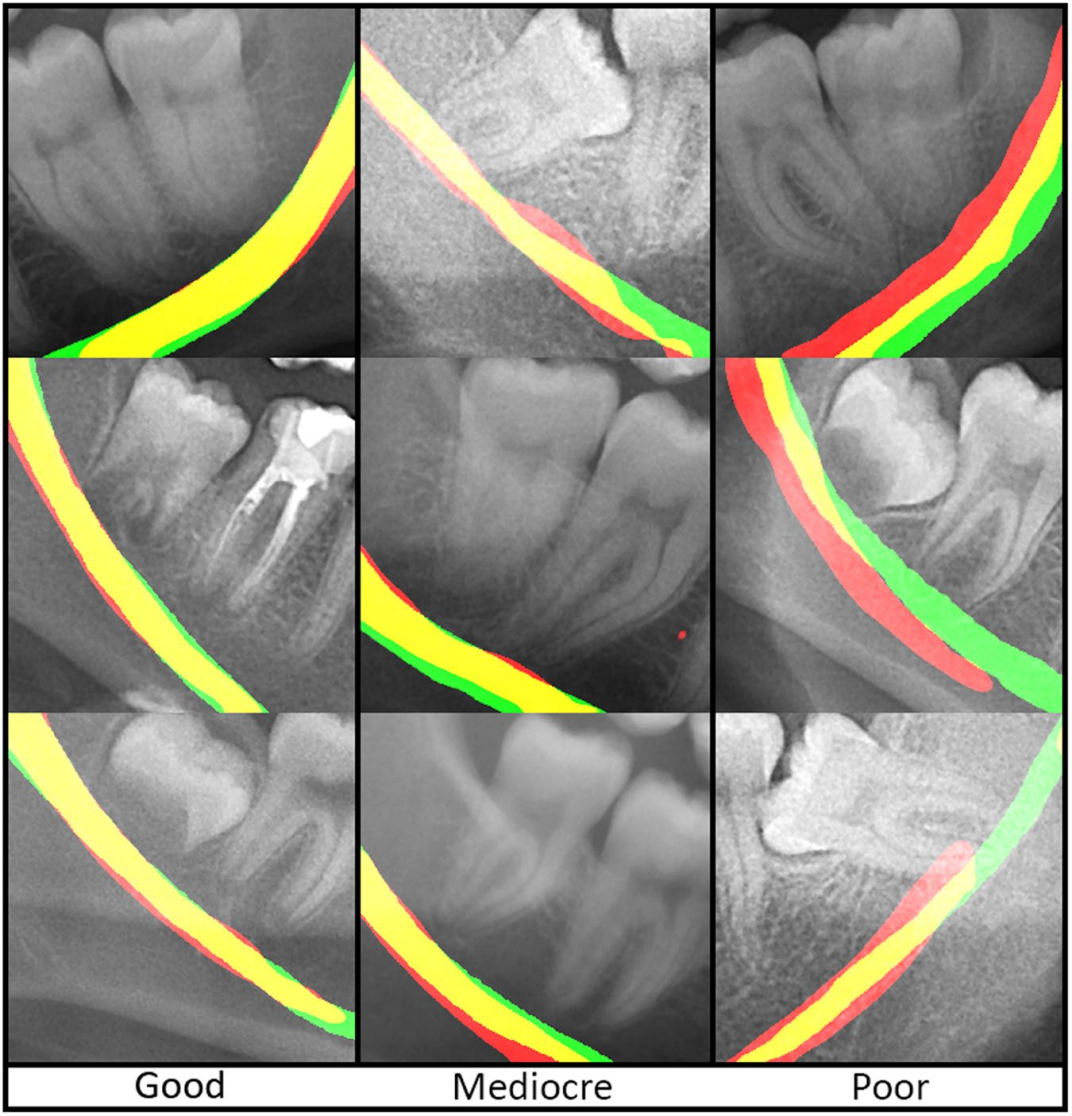
Overview of inferior alveolar nerve segmentations. Green: manual segmentation, red: automatic segmentation, yellow: overlap between automatic and manual segmentation.

In the next step, all segmented teeth and the IAN were transformed to white and the background to black. In this way different Portable Network Graphics (PNG) files were created for the full dentition, and also separately for the lower third molars and the IAN.

The PNGs with all the segmented teeth were used as the gold standard for the training of the deep-learning network used in step 2 to obtain the full dentition. The PNGs with all the segmented teeth (as input) as well as those with the isolated third molars (as gold standard) were used for the first training of step 3. After the training reached its maximum DICE-coefficient^31^ using these data, the network was re-trained by using the result from step 2 in case all present M3’s were detected as an additional data input. For step 5 the network was trained using the manually segmented IANs on the whole OPGs resulting in low accuracy segmentations. The results of the low accuracy IANS from step 5 and M3s from step 3 were cropped in respectively step 6 and 4 and trained in step 7 until the maximum DICE- coefficient was achieved.

### Deep learning training data split

The OPGs and crops were randomly split in a training group (70%) and a validation/test group (30%) prior to the data augmentation. Due to the amount of available scans the validation and test groups were taken as one single group. Data were subsequently checked for unequal distributions of missing third molars and corrected where needed.

### Statistical analysis

The segmentations were assessed by determining the overlap between the gold standard and the deep-learning segmentation using DICE coefficient^31^. The mean, median, standard deviation, and 5–95% percentiles of all the DICE coefficients, Jaccard-indices, sensitivity and specificity were reported for a training and test subset for the M3s (step 3) and the segmented IANs (step 7). The Jaccard index and DICE-coefficient are interchangeable but reported both for convenience^32^.

## Supplementary information

Appendix

## Data Availability

The datasets generated during and/or analysed during the current study are available from the corresponding author on reasonable request.
