# Supplementary material for: Automated detection of third molars and mandibular nerve by deep learning
Source: Sci Rep. 2019 Jun 21;9:9007. doi: 10.1038/s41598-019-45487-3 (PMC6588560; doi:10.1038/s41598-019-45487-3)
Supplement: Supplementary file 1 — Appendix [file 41598_2019_45487_MOESM1_ESM.pdf]

1 Automated detection of third molars and mandibular nerve by deep learning

Shankeeth Vinayahalingam BSc[1,3]

Tong Xi M.D., D.M.D., PhD[1]

Stefaan Bergé M.D., D.M.D., Ph.D[1]

Thomas Maal MSc., PhD [1,3]

Guido de Jong MSc[2]

1. Department of Oral and Maxillofacial Surgery, Radboud University Nijmegen Medical Centre, Nijmegen, the Netherlands.

2. Department of Neurosurgery, Radboud University Nijmegen Medical Centre, Nijmegen, the Netherlands.

3. Radboudumc 3D Lab, Radboud University Medical Center, Nijmegen, the Netherlands

2 Appendix – Details on the segmentation workflow steps

Data pre-processing and data augmentation (Step 1)

For the data pre-processing each OPG of 2048x1024 pixels was scaled to the final resolution of 1024x512 pixels using Matlab 2015a (MATLAB 2015a, The MathWorks, Inc., Natick, Massachusetts, United States). In python (Python Software Foundation. Python Language Reference, version 3.5. Available at http://www.python.org) contrast enhancement using the numpy toolkit (© Copyright 2017 NumPy developers. 2017). The contrast enhancement was applied to the scaled OPGs by determining the mean and standard deviation of the gray-scale pixel values of the whole dataset. The mean is subtracted from each pixel value and subsequently divided by the standard deviation. Data augmentation during deep-learning training is desired to strengthen the invariance and robustness properties of the network (Ronneberger et al. 2015). In this case, the final black and white images were mirrored vertically, horizontally and both at the same time. Four variants off all given images were created in the end to aid with rotation invariance.

Whole dentition and rough IAN segmentation using deep-learning (Steps 2 & 5)

All deep-learning steps were performed using Keras (Chollet 2015) with the Tensorflow (Abadi et al. 2015) backend in python. The full dentition was automatically segmented by a variant of the U-net deep-learning network (Ronneberger et al. 2015). Similarly the rough IANs were segmented. The design as used in this study is shown in Appendix Figure 1. The input was the pre-processed OPG and the output the segmented total dentition. Loss by means of the (negative) smoothed DICE score as the deep learning metric was computed between the deep learning segmentation and the manual segmentation. The network is trained using a default ADAM (Kingma and Ba 2015) optimizer with a learning rate of $1*10^{-4}$ with high momentum β-values (β1 = 0.9, β2 = 0.99) and no decay. Training was stopped after 32 generations have passed.

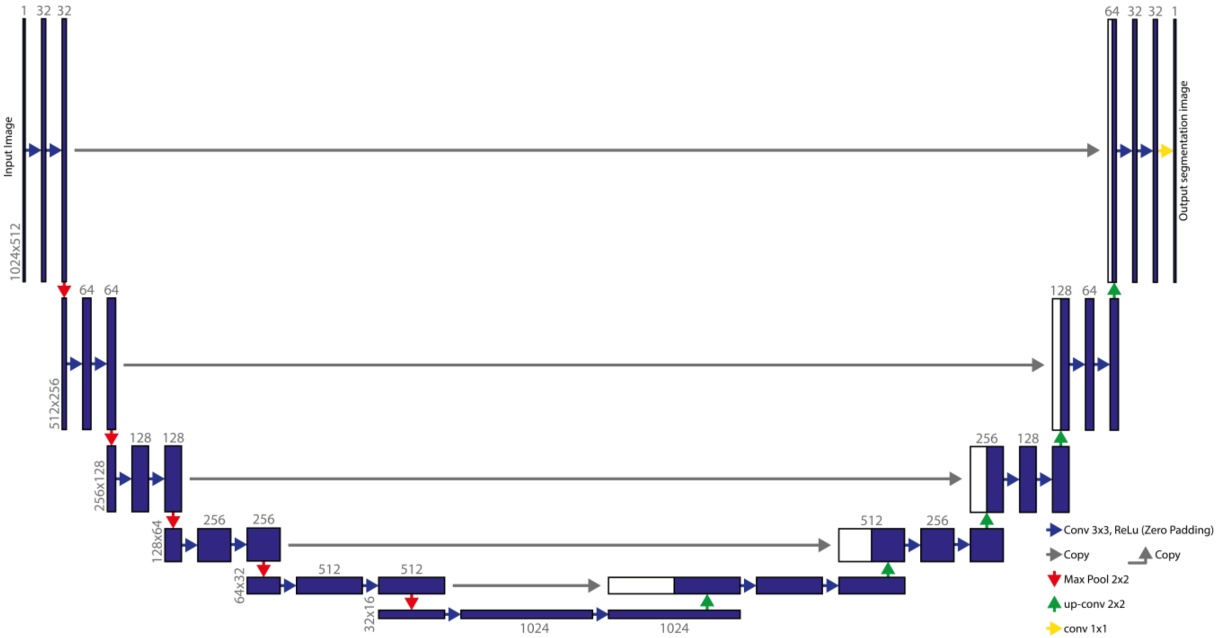

Appendix Figure 1 U-net for full dentition and rough IAN segmentation: These segmentations are executed using a 6-layer U-net with two 1024 kernel-steps in the deepest layer and an implemented zero-padding, since there is no usage of overlap-title strategy.

Lower third molar segmentation using deep-learning (Step 3)

The lower third molar was automatically segmented by a different variant of the U-net deep-learning network. In contrast to the second step a double-input U-net is applied using the pre-processed OPGs and the separately segmented third molars. This starts by two layers of 32-kernel convolution steps per image. The result of these convolutions will be concatenated and used as an input for a similar U-net with the same learning rate, loss and training stopping rules as mentioned in Step 2. The same learning conditions as the whole dentition were used with exception of the learning rate (learning rate=$5*10^{-5}$).

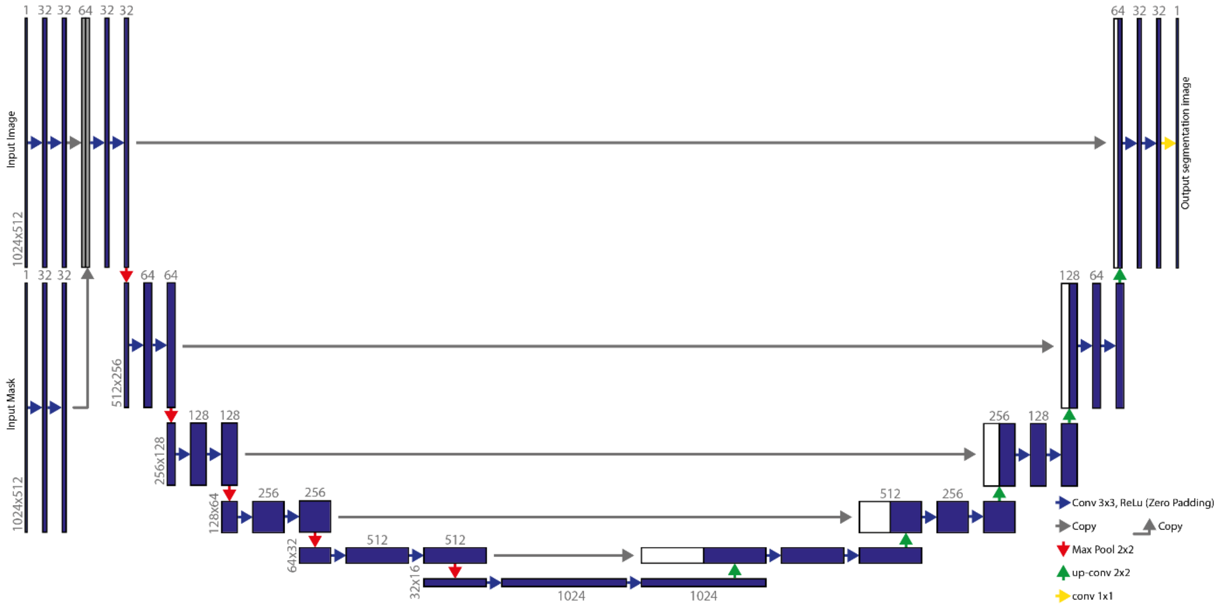

Appendix Figure 2 Double Input U-net: Both input images will undergo two convolution layers with zero padding using 32 kernels per convolution which will be merged to a 64 channel image. Otherwise this U-net is identical to that of Appendix Figure 1.

Cropping the M3 and IAN (Steps 4 & 6)

On each side of the mandibula a crop around the third molar will be made in the original OPG (2048x1024 pixels) based on the computed coordinates of the third molar from step 3. This is similarly executed for the rough IANs obtained from step 5. The coordinates are determined by splitting the image in a left and right section, applying a threshold of 0.5 in the M3 segmentations and detecting the bounding box of the biggest blob in each section. An area of pixels will be used where the centre-top position of the third molar is aligned with the centre-top position of the crop (Appendix Figure 3). This ensures that both the third molar and a part of the IAN are present in the crops.

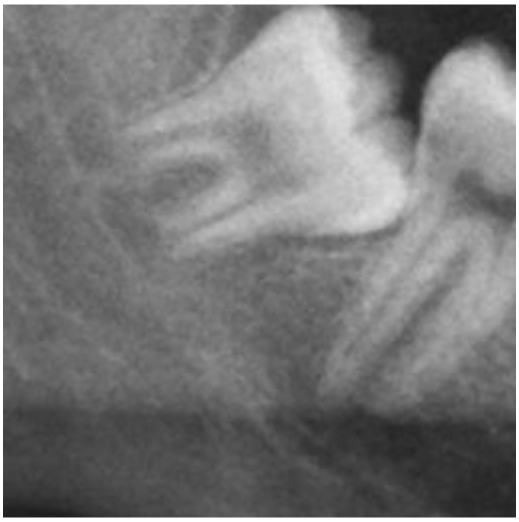

Appendix Figure 3 An automatic crop of a third molar and IAN

IAN segmentation using deep-learning (Step 7)

The IAN was automatically segmented by a similarly structured double input U-net deep learning network as used in step 3. The only differences are that the input of the network does not use 1024x512 pixel images but rather 256x256 pixel images and that a 5-layer U-net was used as a basis. The same training conditions were used identical to those of step 3 with exception of the learning rate (learning rate=$5*10^{-5}$). The cropped images from step 4 and 6 were used as the input for the deep learning network.

3 Legends Tables and figures

Figures

Appendix Figure 1) The structure of U-net used for full dentition segmentation.

Appendix Figure 2) The structure of double input U-net used for third molar segmentations.

Appendix Figure 3) An automatic crop of a third molar and inferior alveolar nerve.
